# MiR-873-5p suppresses cell proliferation and epithelial–mesenchymal transition via directly targeting Jumonji domain-containing protein 8 through the NF-κB pathway in colorectal cancer

**DOI:** 10.1007/s12079-019-00522-w

**Published:** 2019-05-31

**Authors:** Liqiang Wang, Fuquan Jiang, Feng Ma, Bin Zhang

**Affiliations:** 1 Endoscopy Center, China-Japan Union Hospital of Jilin University, Changchun, 130000 Jilin China; 2 Department of Urology, China-Japan Union Hospital of Jilin University, Changchun, 130000 Jilin China; 3 Department of Pathology, China-Japan Union Hospital of Jilin University, Changchun, 130000 Jilin China

**Keywords:** miR-873-5p, Umonji domain-containing protein 8 (JMJD8), Colorectal cancer (CRC), Nuclear factor kappa beta (NF-κB) pathway, Epithelial–mesenchymal transition (EMT)

## Abstract

Colorectal cancer (CRC) is one of the most common leading causes of cancer-related deaths in the world. Recent studies showed that microRNAs (miRNAs) play important roles in the development of diseases, such as CRC. However, the role of miR-873-5p in CRC remains unclear. In this study, we found that miR-873-5p expression was down-regulated in CRC tissues and cell lines, and the down-regulation of miR-873-5p expression was associated with poor survival in patients with CRC. MiR-873-5p could function as a tumour suppressor in CRC. It could inhibit the growth, proliferation, migration and invasion of CRC cells; influence the cell cycle and enhance apoptosis of CRC cells. Bioinformatics and luciferase reporter analyses demonstrated that Jumonji domain-containing protein 8 (JMJD8) was a target of miR-873-5p that could directly target the 3’UTR of JMJD8 and significantly inhibit its expression in CRC cells. This study also verified that JMJD8 functioned as an oncogene in CRC cells. The over-expression of JMJD8 could partly save the harmful effects induced by miR-873-5p in CRC cells, demonstrating that miR-873-5p suppressed carcinogenesis by targeting JMJD8 in CRC. We also verified that miR-873-5p over-expression could suppress CRC cell growth by inhibiting JMJD8 and its downstream NF-κB pathway in CRC. Hence, miR-873-5p inhibited tumour growth, and it may be a potential biomarker and a promising treatment for CRC.

## Introduction

Colorectal cancer (CRC) is one of the most common leading causes of cancer-related deaths in the world (Harrison and Benziger 2011). Although treatment strategies for CRC have improved, the overall survival of patients with CRC after surgical resection remains unsatisfactory (Yi et al. 2016). Hence, the exact pathogenesis of CRC must be illuminated. Many studies have attempted to investigate gene mutation and their products (Li et al. 2015b; Peters et al. 2015; Qu et al. 2017; Szpon et al. 2016), proving that the aberrant activation of signalling pathways is involved in the oncogenesis of CRC (Bodemann and White 2008; Danielsen et al. 2015; Esufali et al. 2007; Sameer et al. 2014). Moreover, the molecular mechanism of CRC metastasis is not fully understood. To facilitate clinical treatment, insight into the molecular mechanism responsible for CRC metastasis is necessary.

Many studies have confirmed that several microRNAs (miRNAs) are closely associated with human tumourigenesis, including cell growth migration and invasion (Zhang et al. 2016). MiRNAs are defined as small non-coding RNAs that are responsible for the post-transcriptional regulation of many genes. To date, numerous studies have demonstrated that dysregulation of miRNAs plays an important role in disease occurrence. For example, Zhang et al. reported that miR-873 inhibits H9C2 cardiomyocyte proliferation by targeting glioma-associated oncogene 1 (GLI1) in congenital heart disease (Zhang et al. 2017). Wang et al. reported that miRNA-873 suppresses glioblastoma oncogenesis and metastasis by inhibiting the expression of IGF2BP1 (Ren-Jie et al. 2015). Furthermore, Cui et al. reported that miR-873 can regulate transcriptional activity of ERα by targeting CDK3 in breast carcinoma cells (Cui et al. 2015). However, the role of miR-873-5p in CRC remains unreported.

Jumonji domain-containing protein 8 (JMJD8) is a member of the JmjC domain-only subgroup, which includes a JmjC domain at 74–269 amino acid residues without other recognisable protein domains (Yeo et al. 2016). Recent studies have demonstrated that JMJD8 is closely associated with angiogenesis and cellular metabolism via interacting with pyruvate kinase M2 (Boeckel et al. 2016). For example, Kok Siong Yeo et al. reported that JMJD8 is an endoplasmic reticulum protein with a JmjC domain (Yeo et al. 2017). Jes-Niels Boeckel et al. reported that JMJD8 regulates angiogenic sprouting or cellular metabolism in endothelial cells by interacting with pyruvate kinase M2 (Boeckel et al. 2016). Furthermore, JMJD8 is required for IKK kinase activation and positively regulates the NF-κB signalling pathway in a TNF-dependent manner (Yeo et al. 2016). However, the role of JMJD8 in cancer remains ambiguous.

In the present study, we first proved that miR-873-5p was down-regulated in CRC and may function as a tumour suppressive miRNA. Functional analysis showed that up-regulation of miR-873-5p significantly suppressed cell viability, proliferation, migration, invasion and EMT process of CRC cells and enhanced the cell apoptosis rate of CRC cells. Furthermore, we demonstrated that JMJD8 acted as a target of miR-873-5p and functioned as the oncogene in CRC progression by activating the NF-κB signalling pathway. Collectively, these results may provide insight into the carcinogenesis mechanisms in CRC.

## Materials and methods

### Patient tissue samples

Sixty pairs of CRC tissues and their adjacent normal tissues from the same patients were collected from the Endoscopy Centre, China–Japan Union Hospital of Jilin University (Changchun, China). Pathological analysis confirmed all the human CRC tissues and their adjacent normal tissues in histology and pathology, and all samples were immediately stored in liquid nitrogen before use. The Ethical Oversight Committee of Endoscopy Centre, China–Japan Union Hospital of Jilin University (Changchun, China) approved this study. The patients’ clinical characteristics are shown in Table 1.

**Table 1:** A correlation analysis between the miR-873-5p expression level and the clinicopathological characteristics

Variables	No. cases(68)	miR-873-5p expression		*p* value
		Low (%)	High (%)	
Age				
<55 years(%)	21	9	12	0.4310
≥55 years(%)	47	25	22	
Gender				
Male	56	25	31	0.0563
Female	12	9	3	
Tumor size				
<5 cm	31	12	19	0.0215
≥5 cm	37	22	15	
TNM stage				
I-II	30	10	20	0.0146
III-IV	38	24	14	
LNM				
No	28	10	18	0.0487
Yes	40	24	16	
Histological type				
Squamous	42	19	23	0.3182
Adenocarcinoma	26	15	11	

**a**χ ^2^ test. *P*-values in bold print indicate statistically significant differences. TNM, Tumor Node Metastasis

### Cell culture

The human CRC cell lines (HCT116, H29, SW620, LOVO and SW480) and a non-tumourigenic human colorectal epithelial cell line (NCM460) were purchased from the American Type Culture Collection (Manassas, VA, USA). Cells were cultured in DMEM (Invitrogen, Carlsbad, CA, USA) supplemented with 10% FBS (Gibco, Grand Island, NY, USA), streptomycin (100 μg/mL) and penicillin (100 μg/mL). Cells were maintained in a humidified atmosphere at 37 °C with 5% CO_2_.

### Transfection assay

Pri-miR-873-5p or antisense oligonucleotides against miR-873-5p (ASO-miR-873-5p) and respective controls including miRNA negative control (pcDNA3) and negative control antisense oligonucleotides (ASO-NCs) were transfected into human CRC cell lines by using Lipofectamine™ 2000 (Thermo Fisher Scientific, Inc.).

### Real-time quantitative PCR (RT-qPCR)

Total RNA was extracted using the Thermo Scientific GeneJET RNA Purification Kit. Quantitative PCR assays of JMJD8, E-cadherin, cytokeratin, vimentin, p65 and p-p65 were performed using SYBR-Green PCR Master Mix (Thermo Fisher Scientific, Inc.) on a VIAA7 qPCR System (Life Technologies). MiR-873-5p levels were also detected via a miRNA-specific TaqMan MicroRNA Assays kit (Applied Biosystems, Foster City, CA, USA). In the present study, sequences of the primers for RT-qPCR were as follows:

JMJD8-F: 5’-ACTTCACTGAGTGGGCATCC-3′.

and JMJD8-R: 5’-GCTGACAGGGCTAGAGATGG-3′;

p65-F: 5’-CAAGCGAGGAGGGGACGTG-3′.

and p65-R: 5’-CCCCCAGAGCCTCCACCC-3′;

GAPDH-F: 5’-CCATGTTCGTCATGGTGTG-3′.

and GAPDH-R: 5’-GGTGCTAAGCAGTTGGTGGTG-3′.

### Cellular proliferation and colony formation

MTT assay was performed to investigate cell viability. Firstly, 3 × 10^3^ HCT116 or SW480 cells that were transfected with miR-NC, pr-miR-873, ASO-NC and ASO-miR-873 were sowed into 96-well plates for 24 h. The cellular proliferation rate was detected at 24, 48 and 72 h using a Quant Universal Microplate Spectrophotometer (BioTek, Winooski, VT, USA) at 490 nm. For the colony formation assay, HCT116 or SW480 cells that were transfected with pr-miR-873, ASO-miR-873 or their relative controls were seeded into 12-well plates (300 cells per well) and cultured for 12 days. Subsequently, crystal violet was used to stain cells, and communities greater than 50 cells were counted.

### EGFP reporter assay

To determine the direct target of miR-873-5p, HCT116 or SW480 cells that were co-transfected with pri-miR-873-5p or ASO-miR-873-5p and the 3’UTR of JMJD8 or the mutant 3’UTR of JMJD8 were cultured into 48-well plates for 48 h. The binding site of miR-873-5p in the JMJD8 3’UTR was mutated, and EGFP activity was measured.

### Flow cytometry

HCT116 and SW480 cells that were transfected with miRNA oligonucleotides were incubated for 48 h and then collected for cell cycle distribution analysis. The cells were washed four times with PBS and fixed in 75% ethanol at 4 °C overnight. Subsequently, the cells were washed again after fixation, incubated with RNase A and treated with PI stain (Beyotime Biotech). A BD FACSCanto™ II flow cytometry system was used to analyse the cell cycle by ModFit LT software package, and annexin V-FITC was employed to evaluate cell apoptosis.

### Transwell migration and invasion assays

In the migration and invasion assays, HCT116 and SW480 cells transfected with plasmids for 24 h were collected and resuspended in serum-free medium. Subsequently, 1.5–2 × 10^5^ of HCT116 and SW480 cells were added to the upper transwell chamber (8 μm pore size, Corning, USA) inserts with or without matrigel (BD Biosciences, USA). Meanwhile, the lower transwell chamber was filled with 30% FBS and incubated for 48 h at 37 °C. Cell migration and invasion were determined by bright-field microscopy.

### Western blot

Antibodies containing E-cadherin, cytokeratin, vimentin, JMJD8, P65, P-P65, BCL-2, BCL-XL, surviving, GAPDH and lamin A were obtained from Abcam (Eugene, OR, USA). RIPA lysis buffer (Sigma, St. Louis, MO, USA) lysed the transfected cells for total protein extracts, including protease inhibitor cocktail (Roche, Mannheim, Germany). The concentrations of total protein were detected by the BCA method (Beyotime). Protein lysates (30 μg/lane) were separated on 10% SDS-PAGE and transferred onto polyvinylidene fluoride membranes. Proteins were incubated with primary antibodies at 4 °C overnight following the manufacturer’s instructions, and membranes were incubated with HRP-linked secondary antibodies (Jackson, USA). Western blot data were quantified by Alpha Innotech (San Leandro, CA, USA) imaging software.

### Statistical analysis

All data shown are presented as the mean ± SD from at least three independent experiments. Statistical analysis was demonstrated by Student’s t test. Fisher’s and chi-square tests were employed to examine differences between the two groups in clinicopathological characteristics. Multiple groups were compared using one-way analysis of variance, followed by Tukey’s post-hoc test for multiple comparisons. *P* values less than 0.05 were determined using two-sided tests and considered statistically significant (*P < 0.05; **P < 0.01; ***P < 0.001; ****P < 0.0001; NS, not significant).

## Results

### MiR-873-5p was down-regulated in CRC

**Fig. 1:**
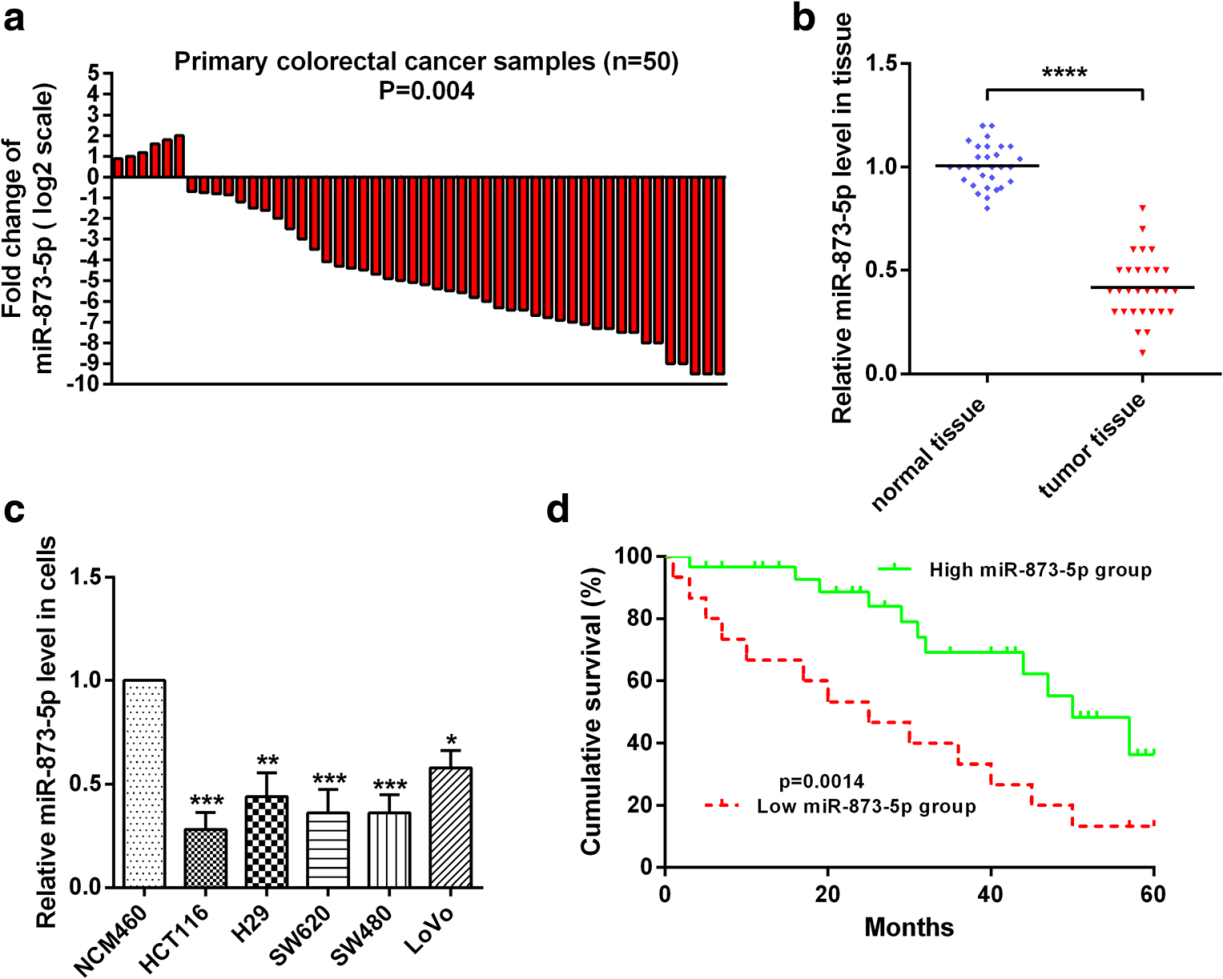
**miR-873-5p was down-regulated in colorectal cancer tissues and cells. a** Total RNA was extracted from colorectal cancer and normal tissues, and then reverse transcribed into cDNA. miR-873-5p levels were showed by RT-qPCR assay. N shows the total number of patients with colorectal cancer. **b** RT-qPCR assay was used to detect miR-873-5p expression levels in colorectal cancer and normal tissues. **c** RT-qPCR assay was used to detect miR-873-5p expression levels in NCM460, HCT116, H29, SW620, LOVO and SW480 cells. **d** Kaplan-Meier survival curves showed that miR-873-5p expression in colorectal cancer patients. All the experiments were repeated more than three times. *, *p* < 0.05; **, *p* < 0.01;***, *p* < 0.001; ****p < 0.0001

To investigate the role of miR-873-5p in CRC, we measured the levels of miR-873-5p in clinical CRC tissues (*N* = 50) and corresponding normal tissues by RT-qPCR. Compared with the normal counterparts, miR-873-5p expression was significantly down-regulated in 44 of the 50 CRC tissues (88%; Fig. 1a). The average expression level of miR-873-5p markedly decreased by almost threefold in the CRC tissues compared with that in normal tissues (Fig. 1b). We also examined the expression levels of miR-873-5p in CRC cell lines and corresponding normal cell line. The results showed that miR-873-5p expression was down-regulated in CRC cell lines compared with that in the corresponding normal cell line (Fig. 1c). To investigate the correlation of miR-873-5p level with survival, Kaplan–Meier survival analysis results showed that patients with CRC and a high miR-873-5p level had a longer overall survival rate than those with CRC and low miR-873-5p expression (*p* < 0.05; Fig. 1d). These data indicated that miR-873-5p levels were negatively correlated with CRC malignancies.

### MiR-873-5p functions as a tumour suppressor in CRC cells

**Fig. 2:**
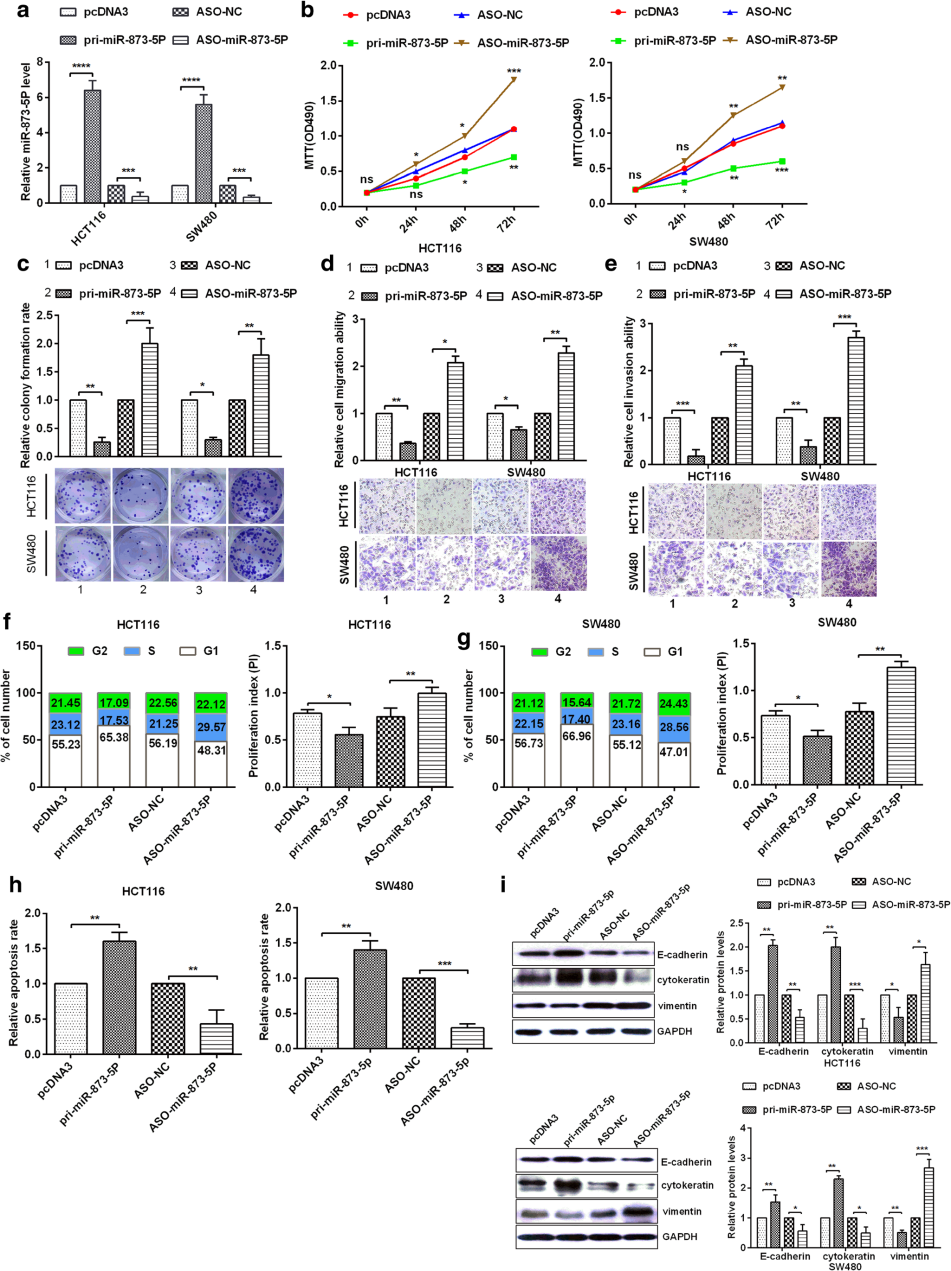
**miR-873-5p inhibits tumorigenesis in colorectal cancer tissues and cell lines. a** The expression of pri-miR-873-5p or ASO-miR-873-5p was analyzed in HCT116 cell or SW480 cell via RT-qPCR. **b** Analysis of miR-873-5p on HCT116 and SW480 cellular viabilities were confirmed by MTT assay. **c** Colony formation ability of HCT116 and SW480 cells were confirmed by colony formation assay. **d**–**e** MiR-873-5p suppressed cell migration ability and cell invasion ability were demonstrated via transwell migration assays or transwell invasion assays. **f**–**g** Flow cytometry assay showed the cell cycle in HCT116 and SW480 cells by transfectting with pri-miR-873-5p or ASO-miR-873-5p and respective controls, and proliferation index (PI) was counted via PI = (S + G2)/G1. **h** The apoptosis rate was demonstrated via Flow cytometry or AV/PI double staining. **i** Western blot assays showed that The protein levels of E-cadherin, cytokeratin, and Vimentin in HCT116 and SW480 cells by transfectting with pri-miR-873-5p or ASO-miR-873-5p and respective controls. All the experiments were repeated more than three times.*, *p* < 0.05; **, *p* < 0.01;***, *p* < 0.001

To investigate whether miR-873-5p affects the progression of CRC, HCT116 and SW480 cells were transfected with pcDNA3, pri-miR-873-5p, ASO-NC or ASO-miR-873-5p. RT-qPCR assays showed that the up- and down-regulation of miR-873-5p in HCT116 and SW480 cells were valid by transfection with the above plasmids or oligonucleotides (Fig. 2a). The over-expression of miR-873-5p dramatically inhibited cell proliferation, and miR-873-5p knockdown significantly increased cell proliferation in HCT116 and SW480 cells on the basis of MTT assays (Fig. 2b) and colony formation assays (Fig. 2c). To determine whether miR-873-5p influences the migration and invasion of HCT116 and SW480 cells, the over-expression of miR-873-5p dramatically inhibited the migration and invasion of HCT116 and SW480 cells, whereas the down-regulation of miR-873-5p markedly increased the migration (Fig. 2d) and invasion abilities (Fig. 2e). To analyse whether miR-873-5p affects CRC cell proliferation, cell cycle experiments demonstrated that miR-873-5p could block the cell cycle and retard G1-S transition in HCT116 and SW480 cells (Figs. 2f, g). Compared with the control group, transfection with pri-miR-873-5p showed a high apoptosis rate, whereas transfection with miR-873-5p inhibitor showed a low apoptosis rate (Fig. 2h). Finally, Western bolt detected the pivotal markers of EMT, such as E-cadherin, cytokeratin and vimentin. The expression levels of E-cadherin and cytokeratin (epithelial marker) markedly increased, whereas that of vimentin (mesenchymal marker) dramatically decreased after transfection with pri-miR-873-5p in HCT116 and SW480 cells (Fig. 2i). By contrast, the expression levels of E-cadherin, cytokeratin and vimentin were reverse when transfected with ASO-873-5p (Fig. 2i). Therefore, miR-873-5p played a tumour inhibition role by suppressing cell proliferation, cell migration and invasion; inhibiting the cell cycle by blocking G1-S transition and enhancing cell apoptosis in CRC cells.

### JMJD8 is a direct target of miR-873-5p in CRC cells

**Fig. 3:**
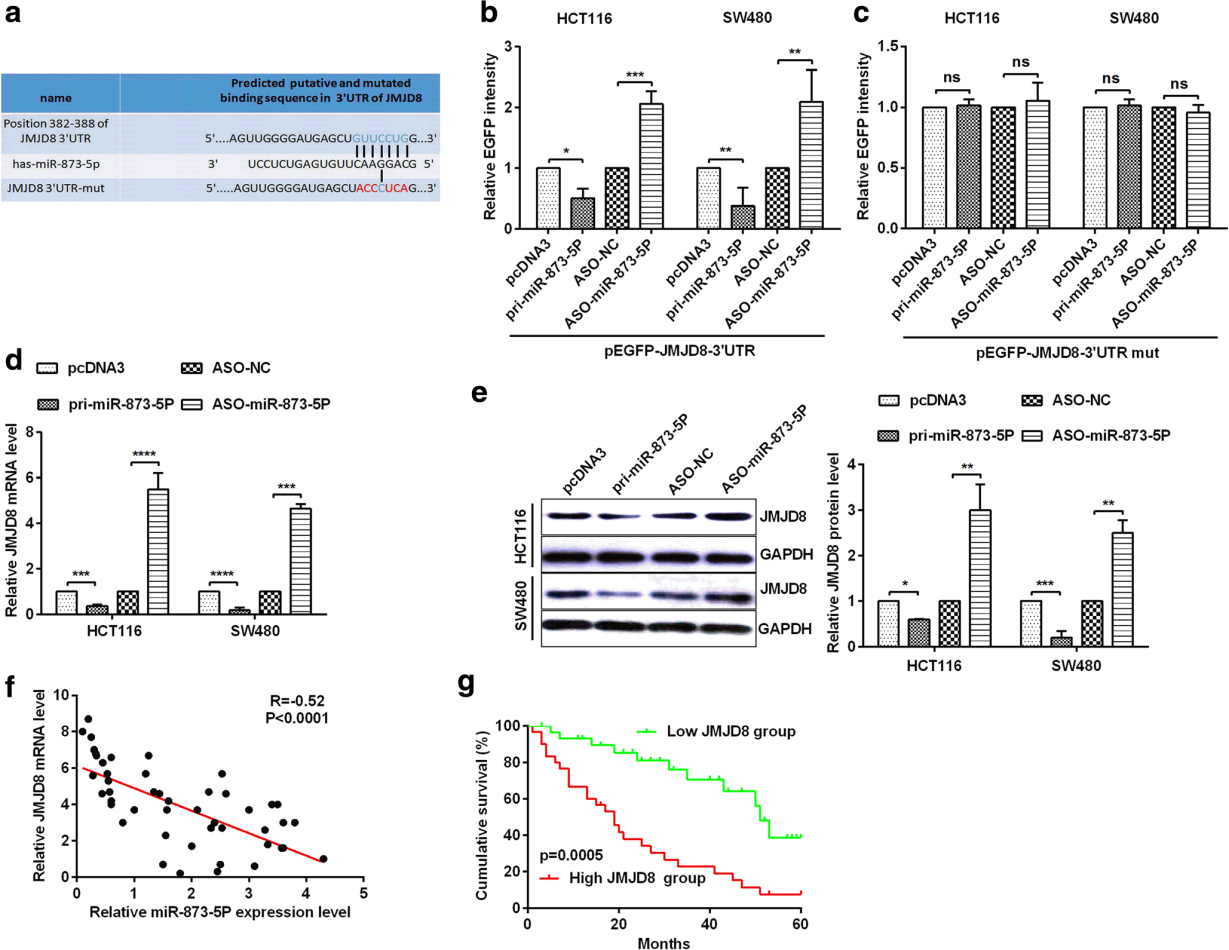
**miR-873-5p directly targets JMJD8 in colorectal cancer cells. a** The predicted miR-873-5p binding sites using TargetScan 7.1 in JMJD8 mRNA 3’UTR or the mutational 3’UTR of JMJD8 mRNA were confirmed. **b**–**c** HCT116 and SW480 cells were co-transfected with pcDNA3/EGFP-JMJD8 3’UTR or 3’UTR-mut and pri-miR-873-5p or ASO-miR-873-5p, and the EGFP intensity was detected via spectrophotometer, which showed that JMJD8 is a direct target of miR-873-5p. **d**–**e** In HCT116 and SW480 cells transfected with pri-miR-873-5p or ASO-miR-873-5p and respective controls, JMJD8 mRNA levels and protein expression were measured by RT-qPCR and Western blot. **f** The correlation analysis of experiments data demonstrated negative correlation between the expression level of miR-873-5p and JMJD8 mRNA level. **g** Kaplan-Meier survival curves showed that JMJD8 expression in colorectal cancer patients. All the experiments were repeated more than three times. *p < 0.05; **p < 0.01; ***p < 0.001; ****p < 0.0001; NS, not significant

To investigate the potential mechanism of miR-873-5p in human CRC cells, we speculated the promising targets of miR-873-5p using miRNA.org, TargetScan 7.1 and RNAhybrid. JMJD8 was chosen as a potential target gene of miR-873-5p (Fig. 3a). To prove that miR-873-5p could directly target JMJD8 mRNA, the EGFP reporter plasmids with the 3’UTR or 3’UTR-mut of JMJD8 were constructed. Co-transfection with pri-miR-873-5p and wild-type 3’UTR of JMJD8 could dramatically inhibit relative EGFP activity, whereas co-transfection with ASO-miR-873-5p and wild-type 3’UTR of JMJD8 could dramatically enhance relative EGFP activity (Fig. 3b). Furthermore, the transfection group with pri-miR-873-5p or ASO-miR-873-5p hardly influenced relative EGFP activity of the mutant JMJD8 3’UTR in HCT116 and SW480 cells (Fig. 3c). To further verify the association between miR-873-5p and JMJD8, we detected endogenous JMJD8 mRNA and protein levels in HCT116 and SW480 cells. Transfection with pri-miR-873-5p significantly reduced the expression of endogenous JMJD8 mRNA (Fig. 3d) and protein (Fig. 3e), whereas transfection with ASO-miR-873-5p markedly increased the expression of endogenous JMJD8 mRNA (Fig. 3d) and protein (Fig. 3e). Additionally, we found a negative correlation between miR-873-5p and the JMJD8 mRNA (Fig. 3f). To investigate the correlation of JMJD8 level with survival, Kaplan–Meier survival analysis results showed that patients with CRC and low JMJD8 level had longer overall survival rates compared with patients with CRC and high JMJD8 expression (*P* = 0.05; Fig. 3g). All the above-mentioned results indicated that JMJD8 was a direct target of miR-873-5p, and it was negatively regulated by miR-873-5p in CRC cells.

### JMJD8 serves as an oncogene in CRC cells

**Fig. 4:**
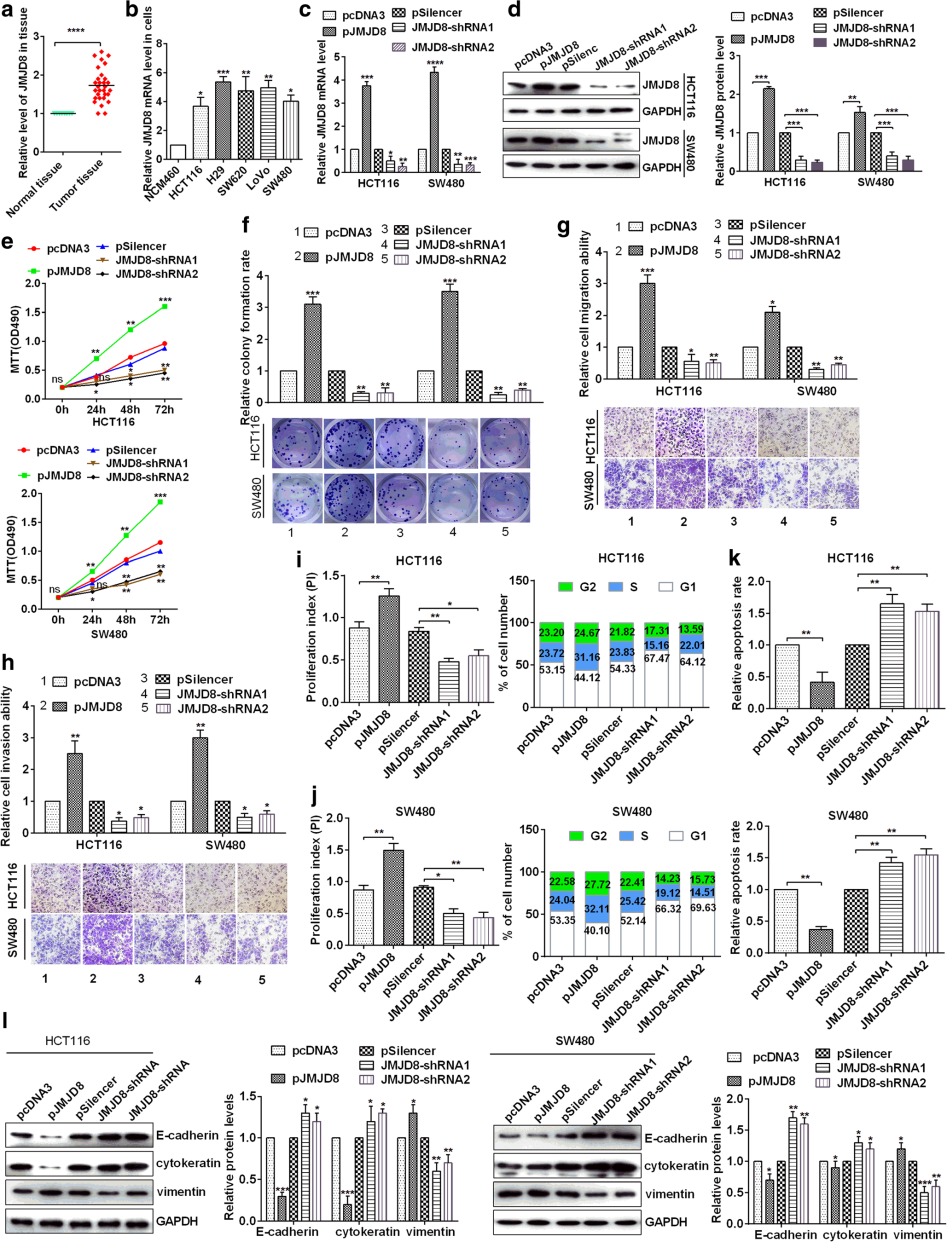
**JMJD8 leads to the development of colorectal cancer cells. a** Relative expression levels of JMJD8 in normal tissues and colorectal cancer tissues were analyzed via RT-qPCR. **b** JMJD8 expression levels in five colorectal cancer cell lines (HCT116, H29, SW620, LoVo and SW480 cells) and a non-tumorigenic human colorectal epithelial cell line (NCM460) were analyzed by RT-qPCR assay. **c**–**d** HCT116 and SW480 cells were transfected with empty vector (pcDNA3), JMJD8 overexpressing plasmid (pJMJD8), or control pSilencer and JMJD8-shRNA1 or JMJD8-shRNA2, and the efficiency of overexpression and knockdown of JMJD8 was demonstrated by RT-qPCR andwestern blotting. **e** Analysis of JMJD8 in HCT116 and SW480 cellular viabilities were confirmed by MTT assay. **f** Colony formation ability of HCT116 and SW480 cells were detected by colony formation assay. **g**–**h** JMJD8 promoted cell migration and cell invasion ability, which were confirmed by transwell migration and invasion assays. **i**–**j** Flow cytometry assay detected the cell cycle in HCT116 and SW480 cells by transfectting with pJMJD8 or JMJD8-shRNA and respective controls, and the proliferation index (PI) was counted via PI = (S + G2)/G1. **k** The apoptosis rate was demonstrated via Flow cytometry and AV/PI double staining. **l** E-cadherin, cytokeratin and Vimentin protein levels in HCT116 and SW480 cells were confirmed by Western blot assays. All the experiments were repeated more than three times. *p < 0.05; **p < 0.01; ***p < 0.001; ****p < 0.0001

To confirm the role of JMJD8 in CRC cells, RT-qPCR assay detected the levels of JMJD8 mRNA in 50 paired CRC tissues and adjacent non-cancerous CRC tissues. The results demonstrated that JMJD8 mRNA levels were significantly up-regulated in CRC tissues compared with those in adjacent non-cancerous colorectal samples (Fig. 4a). In addition, JMJD8 mRNA levels were also up-regulated in CRC cell lines (HCT116, H29, SW620, LOVO and SW480) compared with those in non-tumourigenic human colorectal epithelial cell line NCM460 (Fig. 4b). To investigate whether JMJD8 affects CRC progression, HCT116 and SW480 cells were transfected with pcDNA3, pJMJD8, pSilencer, JMJD8-shRNA1 or JMJD8-shRNA2. RT-qPCR and western blotting were used to detect the levels of JMJD8 mRNA and protein in HCT116 and SW480 cells (Figs. 4c, d). The over-expression of JMJD8 significantly enhanced cell proliferation, whereas JMJD8 knockdown dramatically inhibited cell proliferation in HCT116 and SW480 cells on the basis of MTT assays (Fig. 4e) and colony formation assays (Fig. 4f). To further confirm whether JMJD8 influences cell migration and invasion, over-expression of JMJD8 significantly enhanced the migration and invasion of HCT116 and SW480 cells by transwell migration and invasion assays (Figs. 4g, h). Flow cytometry assay detected cell cycle and apoptosis in HCT116 and SW480 cells, and over-expression of JMJD8 could accelerate the cell cycle, enhance G1-S transition and suppress the apoptosis rate. Meanwhile, knockdown of JMJD8 reversed the above results in HCT116 and SW480 cells (Figs. 4i–k). Finally, Western bolt assays demonstrated the down-regulation of E-cadherin or cytokeratin and up-regulation of vimentin in JMJD8 over-expressed cells. Conversely, knockdown of JMJD8 could promote the expression of E-cadherin or cytokeratin and block the expression of vimentin in HCT116 and SW480 cells (Fig. 4l). All the results showed that JMJD8 functioned as an oncogene in CRC cells.

### MiR-873-5p inhibits the NF-κB pathway by down-regulation of JMJD8 in CRC cells

**Fig. 5:**
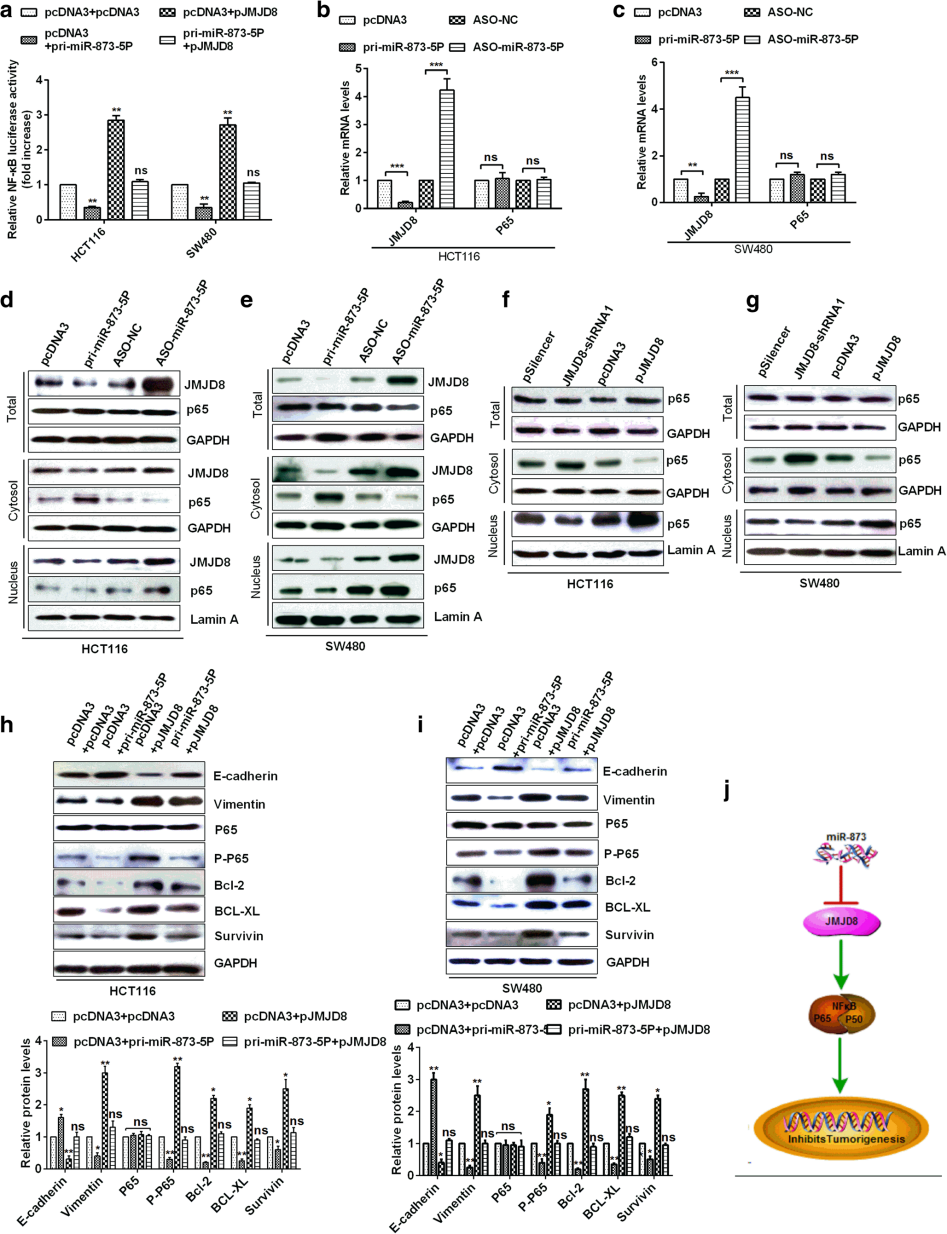
**miR-873-5p inhibits NF-ΚB pathway in CRC. a** NF-κB reporter vector and indicated plasmids were cotransfected into HCT116 and SW480 cells, respectively, after which luciferase activity was measured. **b**–**c** RT-qPCR was used to examine the level of JMJD8, P65 in HCT116 and SW480 cells. **d**–**e** Western blot were used to detect the protein levels of JMJD8 and NF-κB (p65) in the total, cytosol and nuclear cellular fractions of HCT116 and SW480 cells. **f**–**g** Western blot were used to detect the protein p65 in the total, cytosol and nuclear cellular fractions of HCT116 and SW480 cells. **h**–**i** Western blot confirmed the protein level of NF-κB(p65, p-p65), EMT (E-cadherin, Vimentin) and the anti-apoptosis factors level including BCL-2, BCL-XL and survivin in HCT118 and SW480 cells. J Proposed model showed that miR-873-5p supresses tumorigenesis by targeting JMJD8 and inhibiting NF-κB pathway. All the experiments were repeated more than three times. *p < 0.05; **p < 0.01; ***p < 0.001; ****p < 0.0001; NS, not significant

To determine whether miR-873-5p affects the NF-κB pathway in CRC cells, luciferase reporter assays demonstrated that NF-κB transcriptional activity was significantly higher in pJMJD8-transfected cells than in pcDNA3-transfected cells. Moreover, NF-κB transcriptional activity was relatively lower in JMJD8-down-regulated (pri-miR-873-5P-transfected) cells than in pcDNA3-transfected cells. However, NF-κB transcriptional activity in pri-miR-873-5P and pJMJD8 co-transfected cells was the same as in pcDNA3 and pcDNA3 co-transfected cells (Fig. 5a). RT-qPCR assays showed the expression levels of JMJD8 and NF-κB (P65) genes in HCT116 and SW480 cells. JMJD8 expression was suppressed after miR-873-5p over-expression, and the total expression level of p65 did not change after miR-873-5p over-expression (Figs. 5b, c). Western blot analysis demonstrated that JMJD8 expression was suppressed or increased after miR-873-5p over-expression or knockdown, and the total expression level of p65 did not change after miR-873-5p expression changed, and overexpression of miR-873-5p promoted p65 entry into the nucleus, whereas miR-873-5p knockdown inhibited p65 entry into the nucleus(Figs. 5d, e). Is miR-873-5p regulating p65 into the nucleus by regulating JMJD8? Western blot demonstrated that the total expression level of p65 did not change after JMJD8 expression changed, and JMJD8 overexpression promoted p65 entry into the nucleus, whereas JMJD8 knockdown inhibited p65 entry into the nucleus(Figs. 5f, g). Our results demonstrated that miR-873-5p regulated p65 into the nucleus by regulating JMJD8. Therefore, we hypothesised that JMJD8 activated NF-κB in CRC cells. To clarify this hypothesis, we detected the expression levels of E-cadherin, vimentin, p65, p-p65, Bcl-2, BCL-XL and survivin and found that the expression levels of E-cadherin, vimentin, p65, p-p65, Bcl-2, BCL-XL and survivin were contrary in miR-873-5P or JMJD8 over-expressed CRC cells. The expression levels of E-cadherin, vimentin, p65, p-p65, Bcl-2, BCL-XL and survivin were similar in miR-873-5P and JMJD8 co-transfected CRC cells than those in pcDNA3 and pcDNA3 co-transfected CRC cells (Figs. 5h, i). The proposed model showed that miR-873-5p supressed tumourigenesis by targeting JMJD8 and inhibiting the NF-κB pathway (Fig. 5j). These results showed that miR-873-5p inhibited tumourigenesis by directly targeting JMJD8 3’UTR and down-regulating JMJD8 expression, thereby inhibiting the NF-κB pathway in CRC.

## Discussion

CRC is a serious malignancy, which is the main cause of cancer-related deaths all over the world (Qu et al. 2017). Although CRC treatments have significantly improved, the prognosis of patients with CRC is still poor. Therefore, to identify therapeutic targets of CRC, the molecular mechanism of CRC progression must be uncovered (Dowling et al. 2017). Increasing studies have suggested that miRNAs play an essential role in the development of diseases, especially in tumourigenesis (Kloosterman and Plasterk 2006; Lu et al. 2005). Furthermore, miRNAs play an essential role in tumour suppressors and are associated with various biological processes, including tumour initiation and tumour progression in various types of cancers, including CRC (Han et al. 2016; Li et al. 2015a; Liu et al. 2016; Xie et al. 2016; Xuan et al. 2015). However, the function of numerous miRNAs remains poorly understood in many types of cancers. For example, a recent study showed that miR-873-5p can control gastric cancer progression via targeting hedgehog–GLI signalling (Cao et al. 2016). However, the role of miR-873-5p in CRC has not been analysed.

Here, we first exhibited that miR-873-5p was down-regulated in CRC compared with normal tissues. Our results were in agreement with findings of other relevant studies that miR-873-5p was weakly expressed in cancers (Ren-Jie et al. 2015; Zhang et al. 2017). Wang et al. reported that down-regulation of miRNA-873 expression significantly enhances glioblastoma tumourigenesis and metastasis via promoting the expression of IGF2BP1, and the overall survival rate of patients with miRNA-873 down-regulation was dramatically shorter than that of patients with miRNA-873 up-regulation (Ren-Jie et al. 2015). Our results also revealed that the down-regulation of miRNA-873 was associated with poor survival in patients with CRC. Furthermore, we found that miR-873-5p expression was down-regulated in CRC cell lines. In this study, we first proved that the up-regulation of miR-873-5p could suppress the proliferation, migration and invasion of CRC cells, inhibit the cell cycle by retarding G1-S transition and EMT and enhance cell apoptosis. All these effects indicated the tumour inhibition role of miR-873-5p in CRC.

Luciferase assays showed that JMJD8 was a target of miR-873-5p. MiR-873-5p could down-regulate JMJD8 expression via binding to the 3’UTR in CRC. Pearson’s correlation analysis revealed that miR-873-5p was inversely correlated with JMJD8, which was up-regulated when miR-873-5p was poorly expressed in CRC and cell lines. JMJD8 functions as a cancer-causing protein in some cancer cell lines, including endothelial cells (Boeckel et al. 2016). In this study, we proved that JMJD8 served as an oncogene in CRC cells. The over-expression of JMJD8 could partly save the negative effects induced by miR-873-5p in CRC cells, demonstrating that miR-873-5p suppressed carcinogenicity by targeting JMJD8 in CRC.

JMJD8 can activate the NF-κB pathway in endothelial cells (Boeckel et al. 2016). In addition, Liu et al. reported that DCLK1 promotes EMT by the NF-κB pathway in CRC (Liu et al. 2017). We suspect that miR-873-5p also activated the NF-κB signalling pathway in CRC, so we detected the related proteins of the NF-κB signalling pathway. The results indicated that miR-873-5p significantly inhibited the NF-κB pathway via JMJD8 in CRC. RT-qPCR and Western blot assay confirmed that JMJD8 was the upstream signal of the NF-κB pathway in CRC cells. Therefore, miR-873-5p was down-regulated in CRC and cell lines. It inhibited tumours by suppressing JMJD8 expression and inhibiting the inactivation of the NF-κB pathway in CRC.
